# Simulation Study of Helium Effect on the Microstructure of Nanocrystalline Body-Centered Cubic Iron

**DOI:** 10.3390/ma12010091

**Published:** 2018-12-28

**Authors:** Chunping Xu, Wenjun Wang

**Affiliations:** 1 College of Nuclear Equipment and Nuclear Engineering, Yantai University, Yantai 264005, China; 2 College of Engineering, Shanxi Agriculture University, Taigu 030801, China; wwjbuoy03@163.com

**Keywords:** molecular dynamics, Helium effects, crack formation, nanocrystalline BCC iron, mechanical properties

## Abstract

Helium (He) effect on the microstructure of nanocrystalline body-centered cubic iron (BCC-Fe) was studied through Molecular Dynamics (MD) simulation and simulated X-ray Diffraction (XRD). The crack generation and the change of lattice constant were investigated under a uniaxial tensile strain at room temperature to explore the roles of He concentration and distribution played in the degradation of mechanical properties. The simulation results show that the expansion of the lattice constant decreases and the swelling rate increases while the He in the BCC region diffuses into the grain boundary (GB) region. The mechanical property of nanocrystalline BCC-Fe shows He concentration and distribution dependence, and the existence of He in GB is found to benefit the generation and growth of cracks and to affect the strength of GB during loading. It is observed that the reduction of tensile stress contributed by GB He is more obvious than that contributed by grain interior He.

## 1. Introduction

Both displacement damage and production of foreign elements can be introduced in the structural materials under nuclear radiation environment. Point defects and foreign elements generated during neutron irradiation evolve into interstitial clusters, bubbles and voids that lead to severe degradation of mechanical properties, such as hardening, embrittlement, and fracture, void swelling and irradiation creep [1]. Due to extremely low solubility of He in any metal, He as a foreign element in nuclear materials aggregates to form clusters and bubbles near the grain boundary (GB) [2,3] which eventually link up and cause intergranular failure [1,4]. It has been established in theory that the strength of GB is reduced by He or He clusters [5,6,7]. Studies have shown that GB acts as a neutral and unsaturable sink to radiation defects [8,9], or a source, emitting interstitials to annihilate vacancies [10]. Thus, the effects of GB and He on the nuclear material with nanocrystalline structure are widely studied to either understand the mechanisms of radiation effects or solve some practical problems such as the reduction of He embrittlement, void swelling and so on.

Improved radiation tolerance in nanostructure alloy with a high area fraction of GBhas been reported in many experimental studies. A significant grain size effect on radiation tolerance was observed in nanocrystalline body-centered cubic Mo and Fe under He ion irradiation [11,12]. The density and size of defects (dislocation loops, He bubbles) showed a grain size dependence under irradiation [11,12]. The He embrittlement might have been reduced due to the high number density of nanoclusters which acted as effective sinks to radiation defects in nanostructured ferritic alloys (NFS) [13,14]. The behavior of He on microstructure evolution in nuclear materials with GB has been investigated by Molecular Dynamic (MD) simulation and theoretical methods. The study on the nucleation and growth of He clusters in α-Fe GB [15] indicates that the interstitial He atoms tend to combine with nearby monovacancies or He*n*V clusters to form larger He*n*V clusters. The accumulation of He atoms and the evolution of GB structure are affected by local He concentration, temperature, the original GB structure, or their combination in α-Fe [16]. It is demonstrated that the GB tensile strength is reduced by the He segregation which breaks (substitution) or weakens (interstitial) the surrounding interfacial Fe-Fe bonds [5]. The impact of GBs on the clustering properties of He and the possible effect of He on GB decohesion were investigated in references [6,17]. Additionally, the deformation mechanisms in nano-twinned and nanocrystalline materials have been carried out by MD simulations [17,18,19,20], and intergranular crack are reported in those studies.

These studies have provided valuable insights into the He effect on the mechanism of radiation tolerance in nuclear material with GB, but there are few reports about the He effects on the microstructure of nanocrystalline body-centered cubic iron (BCC-Fe). In this work, MD simulations were employed to study the He effects on generation and growth of cracks in nanocrystalline BCC-Fe. The evolution on microstructure of nanocrystalline BCC-Fe under a uniaxial tensile strain condition was investigated through simulated X-ray Diffraction (XRD) patterns. Based on the simulation results, the relationship between the change of microstructure and the degradation of mechanical property during loading is discussed and analyzed.

## 2. Simulation Method

**Figure 1:**
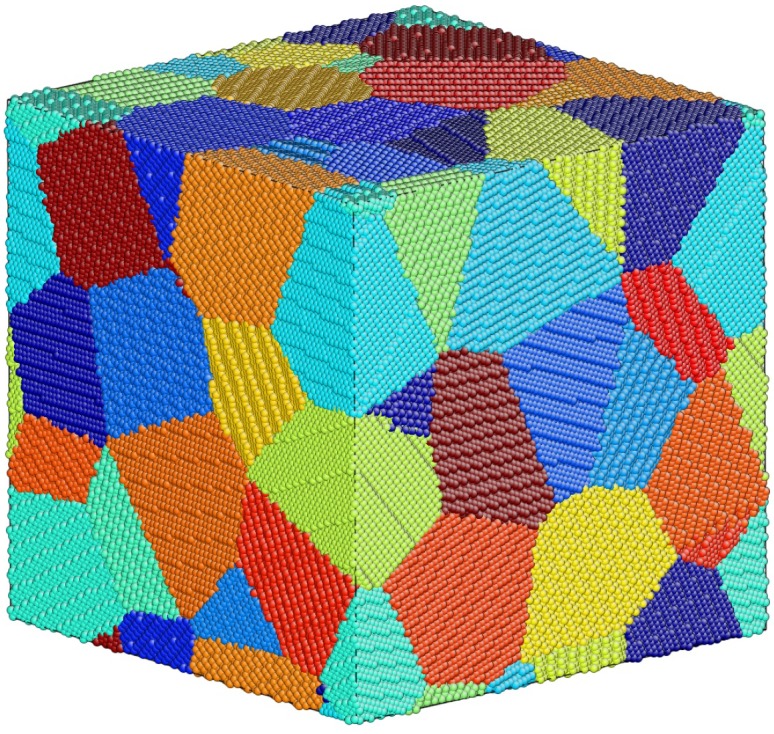
Initial model of nanocrystalline BCC-Fe, the atom was colored according its grain ID (identification).

The model of nanocrystalline BCC-Fe is constructed for simulation using the Voronoi tessellation method [21] with random Euler angles assigned to each grain (see Figure 1). The average grain size of the model with volume of (80×a)^3^Å^3^ is 8.28 ± 0.32 nm. The lattice constant of the model, a, equals 2.8553 Å. The size of grain is represented by the average diameter of grain volume. The simulation is performed using the LAMMPS [22] and the interatomic EAM potential for BCC-Fe developed by Mendelev and his co-worker [23]. Any overlapping atoms that are separated by less than 1.7Å are deleted from the nanocrystalline model and then the final structure with periodic boundary conditions is relaxed using conjugate gradient minimization with an energy tolerance of 10^−5^ eV/Å. There are 40 grains in the resulting structure with 1,001,493 atoms.

**Figure 2:**
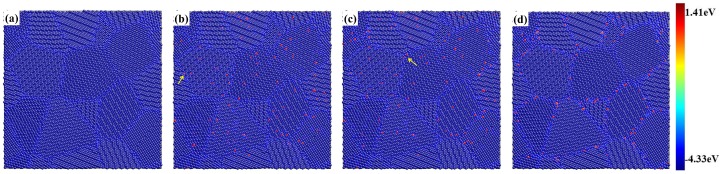
(**a**) Nanocrystalline model after energy minimization; (**b**) The model with BCC He (1%); (**c**) The model with uniform He (1%); (**d**) The model with GB He (1%). The atom in model (**a**–**d**) was colored according to potential energy (see color map). The red ball in (**b**–**d**) represents He atom.

In the picture of pure BCC-Fe model with minimized nanocrystalline structure (Figure 2a), it shows that the potential energy of the iron atom in GB region is slightly larger than that in grain interior region according to the color map. The percentage of iron atoms in the GB region with non-BCC structure, about 18.3%, is calculated by the adaptive common neighbor analysis (ACNA) algorithm. Models of nanocrystalline BCC-Fe with homogeneous and intergranular distribution of He are considered in the present work, because an He atom is introduced randomly in the nuclear structure material under radiation environment, and it tends to form clusters at GBs based on studies from both experiment [11] and theoretical simulation [6]. To clarify the failure of mechanical property contributed by the He distributed at the BCC region, the model doped with He in BCC region is also built for comparison. Substituted He are introduced in the system owing to the fast migration of interstitial He, which are known to strongly interact with vacancies [3,24,25]. According to the distribution and concentration of He, three groups of cubic models are built based on the minimized model (see Figure 2a). Then these nanocrystalline models with He is subjected to the energy minimization again at 0 K.

The distribution of He in three groups of models is as follows: the grain-interior distribution of He is represented by “BCC He”; the homogeneous distribution of He is represented by “uniform He”; and the intergranular distribution of He is represented by “GB He”. The fraction of He distributed in every group of model is 0.5%, 1% and 3%. The picture of the minimized model with the BCC He, the uniform He and the GB He (1%) are shown in Figure 2b–d, respectively. The GB He atom is observed in the model with BCC He or uniform He after the energy minimization (see Figure 2b,c, marked by yellow arrow). In addition, several He clusters are observed in Figure 2b–d since He atoms strongly aggregate into clusters rather than distribute homogeneously [6]. The simulation results about the He effects on the microstructure of polycrystalline BCC-Fe after energy minimization is detailed in Section 3.1.

In order to study the mechanical property of nanocrystalline structure, these models are annealed at 300 K for 10 ps with a Nose/Hoover isobaric-isothermal (NPT), and then subject to a uniaxial tensile load with a strain rate of 10^8^ s^−1^. The simulation step is set at 0.001 ps. The potential of He and Fe-He in references [26,27] are employed in the simulation work. The deformation of models during the process of simulation is visualized by Ovito [28,29]. The evolution on the microstructure of the present nanocrystalline models during loading is described in Section 3.2, and the calculation results is discussed and analyzed in Section 4.1 and Section 4.2.

## 3. Results

### 3.1. He Effects on Microstructure of Nanocrystalline BCC-Fe after Energy Minimization

It is observed in Figure 2b–d that the potential energy of iron atom is changed owing to the introduction of He in nanocrystalline model, so the relative position of single iron atom in the system is modified. In order to investigate the He effect on the microstructure of nanocrystalline after the energy minimization, both the modification of atomic volume distribution and the expansion of lattice constant induced by He are studied. The swelling rate contributed by the expansion of atomic volume and lattice constant are calculated respectively. The atomic volume is represented by the volume of Voronoi cell for lattice site. The lattice constant of present models are obtained through the simulated XRD pattern.

**Figure 3:**
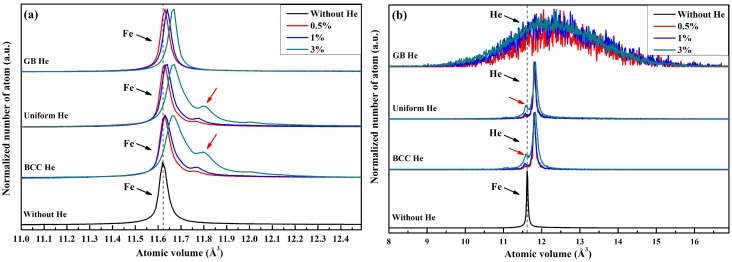
Volume distribution of Fe (**a**) and He (**b**) in polycrystalline model with and without He.

The volume distribution of He and Fe are plotted in Figure 3. Figure 3a shows that the peak volume of Fe in the model doped with BCC He, uniform He and GB He moves right with the increase of He concentration. That is to say the average of atomic volume of Fe is expanded owing to the introduction of He. The small peak marked by a red arrow represents the atomic volume of iron atom affected by nearby He in the grain interior region. It is illustrated that the average distance between two perfect lattice sites which are taken up by iron atoms become further if one of them or its neighbor is replaced by a He.

It is observed in Figure 3b that the average volume of He in the model with uniform He or BCC He is lower than that in the model with GB He. The volume distribution of He in the model with GB He covers a wider volume range compared with that in a model with uniform He. The small peak in volume distribution curves for models with BCC He and uniform He were marked by red arrow in Figure 3b, and these peaks represent the atomic volume of He which formed into He_2_ or He_3_ cluster in the grain interior region after the energy minimization. Figure 3b shows that the average volume of Voronoi cell for lattice site taken up by Fe get larger while these lattice site are occupied by He.

**Figure 4:**
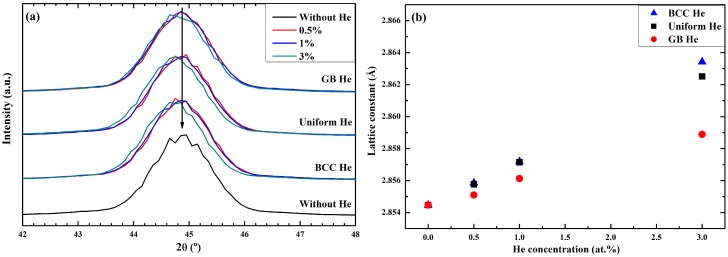
(**a**) Simulated XRD patterns and (**b**) lattice constant of polycrystalline model doped with He.

The existence of He, which occupied BCC lattice site in the models, leads to the distortion or disorder of the surrounding lattice site. In order to study the expansion and distortion of the lattice site of Fe, the XRD patterns of present models are simulated by LAMMPS. According to Bragg formula, the lattice constant of present models are obtained by XRD patterns. The increase of lattice constant (Figure 4b) was observed as a left shift of the {110} peak in XRD patterns (Figure 4a) when the He concentration increases. Figure 4b shows that the expansion of lattice constant caused by the given concentration of He decreases with the decreasing fraction of BCC He.

**Figure 5:**
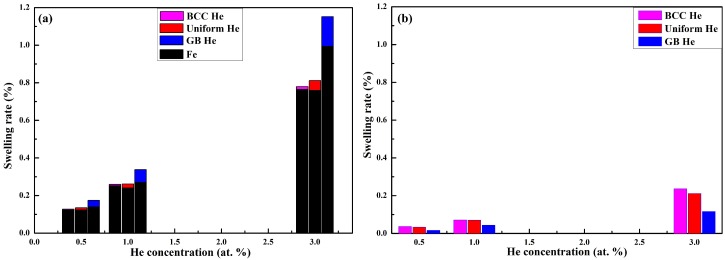
The swelling rate of nanocrystalline models are plotted as a function of He concentration. (**a**) The swelling rate is calculated by the expansion of atomic volume. The swelling rate contributed by Fe is represented by black bar. The swelling contributed by the expansion of He was colored according to the distribution of He in the models; (**b**) The swelling rate is contributed by the expansion of lattice site, the color of the bar represent the distribution of He.

The expansion of atomic volume and lattice constant means that the swelling is unavoidable for the present models doped with He, thus, the swelling rate is calculated after the energy minimization. The total swelling rates contributed by the expansion of the atomic volume are plotted in Figure 5a. The total height of the bar in Figure 5a represents the swelling rate, defined as the percentage change on the average of atomic volume due to the introduction of He in the model. It is observed in Figure 5a that the swelling rate has clearly He concentration and distribution dependence. The total swelling rate of the model increases with the increasing of He concentration, and this value induced by BCC He or uniform He is lower than that induced by GB He. The swelling rate contributed by the atomic volume expansion of He has the same tendency as the total swelling rate. Thus, the expansion of model with given He concentration will continue to increase if the He which is distributed at the BCC region diffuses into the GB region. The swelling rate contributed by the change of lattice constant is shown in Figure 5b as a comparison. It is observed in Figure 5b that the swelling rate obtained by the expansion of lattice constant is slightly lower in the model with GB He than in models with uniform He or BCC He while the total He concentration remains unchanged.

It is clearly observed in Figure 5 that the swelling rate obtained by XRD patterns is lower than that obtained by the expansion of atomic volume. The reason is that the peak position of XRD is determined by the lattice site of BCC Fe atom rather than by the disordered Fe atom which lost the crystal structure. That is to say, the distortion of BCC lattice site (such as disordered Fe) is more severe in the models doped with GB He than in the models doped with BCC He or uniform He. Thus, it is observed that the swelling rate contributed by the expansion of lattice constant is smaller in the former than in the latter. This implies that the volume expansion of present models contributed by both doped He and disordered Fe is larger than that contributed by the expansion of lattice constant. It is deduced that the total swelling rate of present models is contributed by doped He, disordered Fe and BCC Fe. Additionally, the GB structure of nanocrystalline is changed more severely in the model with GB He than in the models with uniform He or BCC He, since the disordered Fe and the GB He is wholly located at the GB region of the former.

### 3.2. He Effect on the Generation and Growth of Cracks in Nanocrystalline BCC-Fe during Loading

**Figure 6:**
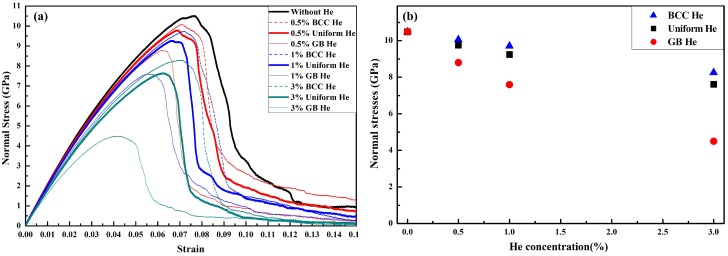
(**a**) Normal stress-strain curves for moles with different He concentration and distribution under uniaxial tensile strain; (**b**) The maximum normal stresses of models with BCC He, uniform He and GB He.

The effect of He on the mechanical property of nanocrystalline BCC-Fe is investigated by LAMMPS. Figure 6a shows the curve of normal stress along the loading direction as a function of strain for models with different He concentration and distribution. It is observed that the peak value of normal stress decreases with increasing He concentration in the model with BCC He, and this value is further reduced if some or all of the BCC He diffuses into the GB region. It is shown in Figure 6a that the tensile strength of nanocrystalline models is also induced by BCC He (see dashed line and blue triangle), because the microstructure is changed in the BCC region (see Figure 3 and Figure 4). It is observed in Figure 6b that the tensile strength of the model with 3% uniform He is close to that of the model with 1% GB He, that is to say, the effect of additional 2.4% uniform He or 0.4% GB He on the model with 0.6% GB He is almost same. It is implied that the reduction of tensile strength is less in the model with a larger fraction of He distributed at BCC region while the He concentration remains unchanged. Moreover, the absence of strain hardening in the present models with He is observed because the normal strength of the present nanocrystalline BCC-Fe is mainly affected by grain size [20,30].

**Figure 7:**
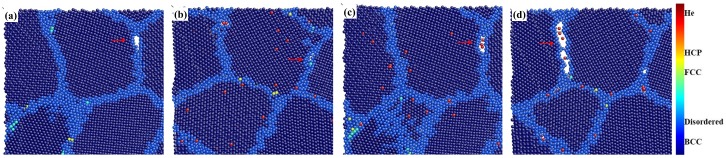
Cracks formed in the model (**a**) without He; (**b**) with BCC He (0.5%); (**c**) with uniform He (0.5%) and (**d**) with GB He (0.5%). The structures of Fe atom analyzed with the help of ACNA algorithm are colored according to the color map, and the red ball represents He atom.

The deformation processes in the present models are visualized and the crack is observed in the GB region. Figure 7a–d show one of the crack appeared at 6.5% strain in the cross section of nanocrystalline model without He and with BCC He, the uniform He and GB He, respectively. Figure 7 illustrates that the crack is formed in the intergranular region during loading [17,31]. The structure of lattice site for atom in models is analyzed with the help of ACNA algorithm. It is observed that the atom of Fe with the structure of FCC or HCP formed into clusters along the GB during the deformation of the model, and those phase transitions are studied in Ref. [19]. The fraction of FCC (face-centered cubic) and HCP (hexagonal close-packed) Fe reaches the maximum at the peak of normal stress and then decreases with the increase of deformation. The existence of He is observed inside the crack shown in Figure 6c,d.

**Figure 8:**
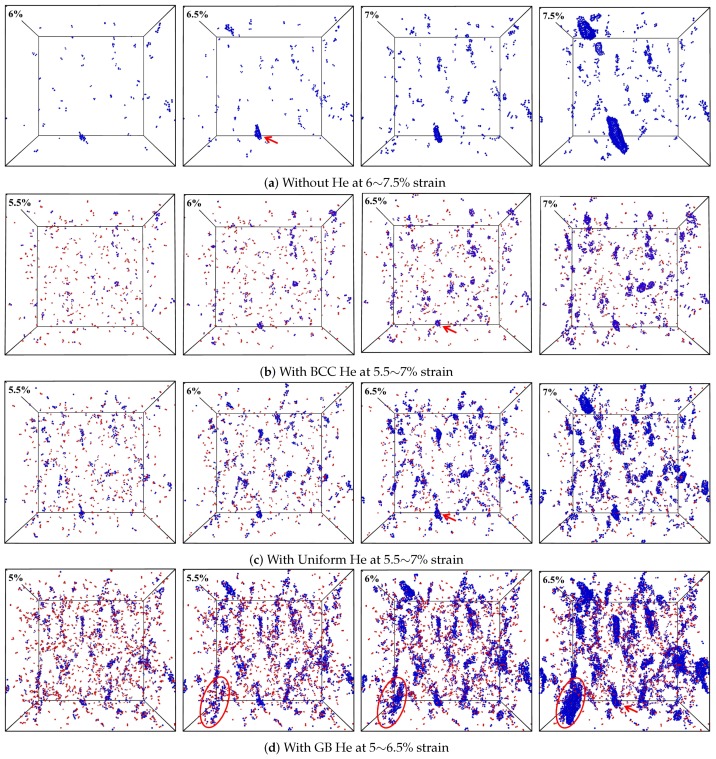
The evolution process of crack in model (**a**) without He at 6∼7.5% strain; (**b**) with 0.5% BCC He at 5.5∼7% strain; (**c**) with 0.5% uniform He at 5.5∼7% strain; (**d**) with 0.5% GB He at 5∼6.5% strain.

Basically, the crack is formed due to the aggregation of vacancies in the GB region during the deformation of present models, and then the Fe with atomic volume greater than 16 Å^3^ (this is the biggest atomic volume in the model without He before loading) is selected to track the generation and growth of crack. The evolution process of the He- and Fe-clusters’ dependence on strain is shown in Figure 8 where the single He and selected Fe atoms are filtered out for clarity. The red arrow in Figure 8 labels the crack shown in Figure 7, and the viewpoint and size of the picture panels are same. If the size of cluster formed from the selected Fe atoms meets a certain scales during loading, it means the crack is opened. It is observed in Figure 8 that the neighboring clusters of He and selected Fe labeled by the oval is gradually linking up and forming bigger cluster or crack [1,4] with increasing deformation of the model. The size of the crack or clusters of selected Fe is less in the model without He than in the model with He. The crack generation sources (and the crack sizes) are more (and larger) in the model with GB He than in the model with BCC He or uniform He for the given He concentration. This shows that He atoms introduced in nanocrystalline BCC-Fe can facilitate the crack formation and growth, consistent with previous results by others [2,6,17].

**Figure 9:**
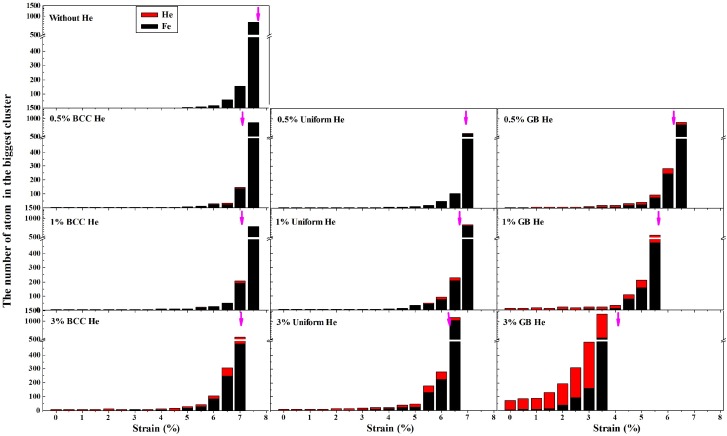
The size of the largest cluster at different He concentration during the deformation of models. The height of bar represents the size of the cluster, consisting of He (red) and Fe (black).

Figure 9 shows the size of the biggest cluster which is formed from the selected Fe and He during the deformation of models where the magenta arrow represents the strain at which the normal stress reached the maximum value. It is observed in Figure 9 that there is a cluster formed by the selected Fe atom in the model with 3% GB He before the tensile stress is loaded. Suppose that there is about 20% of the doped He in the GB region for the model with uniform He. It is shown in Figure 8 and Figure 9 that the size of the cluster grows bigger and the number of selected Fe in the cluster gets larger with increasing fraction of GB He in the deformation of models. These results indicate that the failure of tensile property is promoted by GB He which trends to form He cluster and benefit the crack generation. Additionally, the ratio of He to vacancy also can affect the generation and growth of crack [17].

## 4. Discussion and Analysis

### 4.1. The Simulated XRD Patterns of Nanocrystalline Model during Loading

The failure of tensile strength shown in Figure 6 and Figure 7 was induced by the change of nanocrystalline structure (shown in Figure 3 and Figure 4) which was modified by the introduction of He. It was indicated in Figure 8 and Figure 9 that the degradation of mechanical property was mainly affected by the GB He. In order to further interpret the effect of GB He, uniform He and BCC He on the mechanical property of nanocrystalline BCC-Fe under a uniaxial tensile load, the connection between the strain–stress curves and the corresponding change of microstructure are discussed with the help of the simulated XRD patterns.

**Figure 10:**
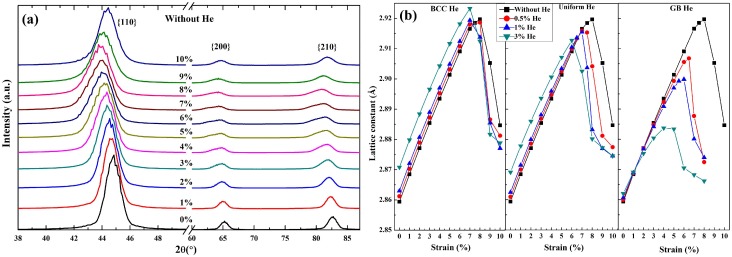
(**a**) Simulated XRD patterns of pure nanocrystalline BCC-Fe at the strain range from 0% to 10%. (**b**) The lattice constant of models under the tensile stresses are plotted as a function of strain.

Figure 10a shows a set of simulated XRD patterns during the deformation of model without He. As the increase of deformation, the {110} peak in the model shifts to left till reaching the maximum movement at ∼8% strain, and then moves back gradually. The lattice constant of nanocrystalline model are obtained by a set of the simulated XRD patterns and plotted as a function of strain in Figure 10b. It is observed in Figure 6a and Figure 10b that the variation of lattice constant is same as that of normal stress with the increase of deformation. So there are some connections between the generation of crack and the expansion of lattice constant.

The degradation of mechanical properties without the effect of He is analyzed firstly. It is observed in Figure 10 (see XRD patterns and the black square) and Figure 6a (see black solid line) that the more variation of the lattice constant is obtained by XRD patterns, the bigger tensile stress is loaded the deformation of the model. The variation of lattice constant is caused by the deformation of single grain whose shape is changed in turn during the loading process of nanocrystalline models. As is shown in Figure 10b (see the black square) and Figure 6a (see black solid line), the decrease of average lattice constant of single grains begins at the maximum loading. However, the crack is generated before the maximum loading is reached (see Figure 7), so there would be a recovery of lattice constant in the grain around this crack. That is to say, the dominant role is acted by the increase of lattice constant before the maximum loading and by the decrease of that after the maximum loading. Therefore the recovery of lattice constant indicates the generation of crack, which starts to appear at GB region while the ultimate strength of the GB is reached. Generally, the smaller ultimate strength of GB means the lower tensile stress needed for the generation of crack.

The role of He played in nanocrystalline model is discussed secondly. It is observed in Figure 10b that the maximum lattice constant is decrease with the increasing concentration of GB He or uniform He, and is changed slightly in the models with BCC He. It is clearly indicated that the maximum lattice constant is variated with the distribution and concentration of He doped in the models. The uniform He have a combination effect of GB He and BCC He on the microstructure of present models during loading. So the role of BCC He and GB He on the expansion of lattice constant and the strength of GB are analyzed as follows respectively.

1. BCC He. It is not difficult to understand that the number of BCC Fe atom per unit volume is reduced in grain interior region due to the BCC He is doped into the nanocrystalline models. Suppose that the lattice is a bunch of springs, which can be broken because of the existence of substitution He. The length of this bunch of springs may need to extend more to balance the given tensile stress if some of the springs are broken before loading. So the maximum lattice constant of model with the concentration of BCC He at 0.5% or 1% is changed slightly comparing that of the model without He. It is also observed that the maximum lattice constant is lower in the model without He than in the model with 3% BCC He (Figure 10), although the tensile strength of the former is stronger than that of the latter (Figure 6b). It is implied that the expansion of lattice constant is unsaturated when the ultimate strength of GB is reached in the model without He. Then the maximum lattice constant during loading is determined by the number of BCC Fe atom per unit volume if there is only BCC He in the model. The maximum of tensile stress in the models with BCC He is reduced because the GB strength is weakened by He or He clusters, which trapped by GB region during energy minimization (see Figure 2) or deformation of the models.
2. GB He. The maximum lattice constant of the models with GB He and the ultimate strength of GB decrease with the increasing concentration of GB He, because the ultimate strength of GB is weakened by GB He and the expansion lattice constant is limited correspondingly. Thus, it is deduced in Figure 10b that the failure of mechanical property is caused due to the ultimate strength GB is reached. It implied that the expansion of lattice constant is governed by the strength of GB while the GB He is introduced in the nanocrystalline models.

According to the above analyses, it is concluded that (1) the tensile strength of nanocrystalline model is mainly determined by the GB strength, which is weakened by He distributed at GB region; (2) the number of BCC Fe atom per unit volume is reduced by He distributed at grain interior region.

### 4.2. The Volume Expansion of Nanocrystalline Model during Loading

The atomic volume of Fe defined as the volume of Voronoi cell for lattice site is changed with the separation of atoms in the models. The work must be done against the atomic interactions to change the distance between atoms in the system. So there is a link between the work done by tensile stress and the swelling of atomic volume of Fe during the deformation of models. In the following discussion, the expansion of atomic volume is considered as a process of energy absorption in the system, or as the result of work done by the stress loaded on the Fe atom. For example, the expansion of atomic volume (see Figure 3a) contributed by Fe is more, the potential energy is larger in the system. The expansion of atomic volume (Figure 3a) means that some certain stresses have been loaded on the models with He before loading. That is to say, the introduction of He in the nanocrystalline Fe is equivalent to doing work on this model. Based on the above description, the swelling and the average of atomic volume are calculated respectively to clearly describe the effect of GB He and BCC He on the present models during loading.

**Figure 11:**
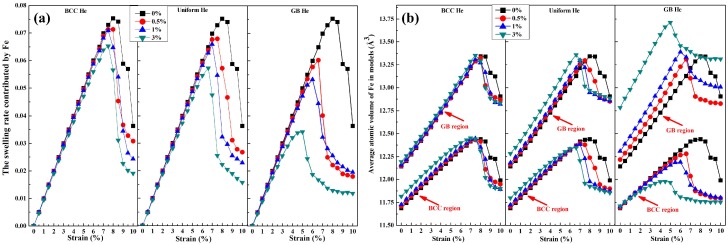
(**a**) Swelling rate contributed by Fe, and (**b**) average atomic volume of Fe in BCC and GB region. Atomic volume of Fe which surround the crack is set as 16 Å^3^, in order to deduct the space took up by the crack.

The swelling rate of present models during loading shown in Figure 11a is contributed by the expansion of atomic volume of Fe. Figure 11a shows that the swelling rate as the function of strain follows the same trend as the tensile stress while the distribution of He is unchanged, and the maximum swelling is reached when the maximum normal stress is loaded on the nanocrystalline models. The average atomic volume of Fe in the models is divided into two parts, GB region and BCC region. The variation of atomic volume as a function of strain in those regions is marked by red arrow as is shown in Figure 11b. It is observed in Figure 11b that the average atomic volume of Fe is lower in BCC region than in GB region for the model without He. It is obvious that the crack has generated in GB region after the maximum volume of Fe is reached in BCC region or GB region. It is illustrated in Figure 11b that the GB region is a weaker area when compared with BCC region due to the ultimate strength of GB is reached earlier than that of BCC region during the loading process. So the tensile strength of nanocrystalline BCC-Fe doped without He is controlled by the GB strength. This point is agreed with the conclusion in Section 4.1.

It is observed in Figure 11b that the difference value of the maximum atomic volume between the GB region and the BCC region get larger with the increase fraction of He distributed in GB region when the He concentration is unchanged. It is implied that the concentration of stress in GB region is enhanced by the He distributed at GB region during the deformation of these models. So the failure of mechanic property of the present models can be concluded as following: (1) the crack generation is not started until the ultimate volume of single Fe is reached for the model without He; (2) the average of atomic volume of GB Fe is affected by GB He, and that of BCC Fe is affected by BCC He; (3) The maximum swelling rate of the atomic volume during loading is mainly limited by the expansion of atomic volume of GB Fe. From the perspective in this section, it is easy to find that the change of lattice constant and the atomic volume during loading contribute to understand the degradation of mechanical property in the nanocrystalline models without He or with He.

Based on the analysis above and the previous studies [5,6,7,17], we conclude that the introduction of He in GB region is a fatal factor for degradation of tensile property in nanocrystalline BCC-Fe comparing with that in BCC region [4]. On condition that the He concentration in models remains unchanged, then the more fraction of He is distributed in the GB region, the more severe degradation of mechanical property is occurred. The mechanical property of nanocrystalline BCC-Fe will be improved if the weaker one of GB and BCC region is strengthened, and the degradation of tensile property will be reduced if the radiation damage on the weaker one is avoided or decreased.

## 5. Conclusions

In summary, He effects on generation and growth of cracks in nanocrystalline BCC-Fe are studied in the present work. The modification of microstructure caused by the He distributed in the nanocrystalline is analyzed by the change of simulated XRD patterns and the atomic volume. For the nanocrystalline BCC Fe with the average grain size of 8.28 ± 0.32 nm, the following conclusions are obtained:

1. The swelling of the model with He is contributed by doped He, disordered Fe and BCC Fe; the He distributed uniformly in nanocrystalline structure causes more expansion of lattice constant than that distributed at grain boundary (GB) structure; the total swelling rate induced by uniform He is lower than that induced by GB He.
2. The tensile strength of nanocrystalline BCC-Fe is reduced after the introduction of He; the strain–stress curve shows He concentration and distribution dependence; the crack generation and growth are observed in the GB region during loading.
3. The tensile strength is affected by the strength of GB which is mainly weakened by He distributed at GB region; the change of lattice constant and the atomic volume during loading should contribute to understand the degradation of mechanical property in the nanocrystalline models.
4. The mechanical property of nanocrystalline BCC-Fe under an irradiation environment would be improved by enhancing the GB strength or reducing He concentration in GB region.

## Abbreviations

The following abbreviations are used in this manuscript:

BBC	Body-centered cubic
XRD	X-ray Diffraction
GB	Grain boundary
ACNA	Adaptive common neighbor analysis
